# Plant Science View on Biohybrid Development

**DOI:** 10.3389/fbioe.2017.00046

**Published:** 2017-08-14

**Authors:** Tomasz Skrzypczak, Rafał Krela, Wojciech Kwiatkowski, Shraddha Wadurkar, Aleksandra Smoczyńska, Przemysław Wojtaszek

**Affiliations:** ^1^Faculty of Biology, Department of Molecular and Cellular Biology, Adam Mickiewicz University in Poznań, Poznań, Poland; ^2^Faculty of Biology, Department of Gene Expression, Adam Mickiewicz University in Poznań, Poznań, Poland

**Keywords:** plants biohybrids, plants communication, tropisms, biological modeling, long distance signaling

## Abstract

Biohybrid consists of a living organism or cell and at least one engineered component. Designing robot–plant biohybrids is a great challenge: it requires interdisciplinary reconsideration of capabilities intimate specific to the biology of plants. Envisioned advances should improve agricultural/horticultural/social practice and could open new directions in utilization of plants by humans. Proper biohybrid cooperation depends upon effective communication. During evolution, plants developed many ways to communicate with each other, with animals, and with microorganisms. The most notable examples are: the use of phytohormones, rapid long-distance signaling, gravity, and light perception. These processes can now be intentionally re-shaped to establish plant–robot communication. In this article, we focus on plants physiological and molecular processes that could be used in bio-hybrids. We show phototropism and biomechanics as promising ways of effective communication, resulting in an alteration in plant architecture, and discuss the specifics of plants anatomy, physiology and development with regards to the bio-hybrids. Moreover, we discuss ways how robots could influence plants growth and development and present aims, ideas, and realized projects of plant–robot biohybrids.

## Introduction

Plants are sessile, autotrophic, eukaryotic organisms that are crucial components of almost every ecosystem and provide multiple resources to humans. Since the Neolithic era, humans have struggled to improve plants, to adapt them to better address their needs. Recently, farming technologies and breeding practices, which include molecular biology and genomics, have been greatly developed (Kanchiswamy et al., 2015; Poland and Rutkoski, 2016). Robots and advanced biosensors are already to use in the agriculture. The development of biohybrids, which are composed of biological and robotic parts, could become a smooth continuation and the next step (Gund, 2015; Ledford, 2017). There is also a growing understanding of biological life that pushes toward utilization of the advantages of combining living organisms and robots within one “being.” Is it feasible to increase the survival rate of cultures, crop yield, and even extend plants “functionalities” by creating biohybrids? The motivation coming from the rising food demand, the spread of great metropolis, and environmental challenges, began the struggle for the creation of robot–plant biohybrids, which will benefit from the synergy between both biological and artificial parts. For crops, biohybrids will help with biotic and abiotic stress management: robotic components could provide the plants with additional sensing, communication, or increased survival capabilities. Moreover, robot–plant biohybrids might become valuable elements of our urban space, even as a promise of living buildings, or at least as an inspiration for using continuous building material (Heinrich et al., 2016, unpublished manuscript^1^).

Robotic prostheses are examples of biohybrids where feedback communication loops take seconds. After that, human motor or sensual capabilities are restored, the feedback is visible, and corrections can be implemented (Halloy et al., 2013; Bensmaia, 2015). It is worth to underline that many other investigated biohybrids systems exploit nanotechnology and soft robotics as tools, which allow them to efficiently interact with a living cell or an organism (Sicard et al., 2010; Ricotti and Menciassi, 2012; Patino et al., 2016). In robot–plant biohybrids, the most intriguing is the lack of nervous system and motor skills, although only a few fast motor plant reactions resembling animals motions are known. The motions of mimosa (*Mimosa pudica*), *Venus flytrap* (*Dionaea muscipula)*, pea’s (*Pisum sativum*) tendrils are good examples of that and have been noticed by engineering due to their interesting mechanism of enwinding (Gerbode et al., 2012; Guo et al., 2015). However, despite being driven by different mechanisms than animals, behavior, decision-making, and even learning, are phenomena that exist in plants (Cvrčková et al., 2016; Gagliano et al., 2016; Schmid, 2016). It has been reported that cognition is restricted not only to brain-having organisms and that some of the complex molecular interactions seem to give a sufficient foundation for non-neuronal cognitive processes (Calvo and Baluška, 2015; Baluška and Levin, 2016). An escape from an illuminated area is an example of behavior that is similar, even on the level of molecular mechanism, in an animal *Caenorhabditis elegans*, with its primitive nervous system, and in roots of *Arabidopsis thaliana*, which lack nervous system (Yokawa and Baluška, 2015). The more general overview, where biorobotics and plant associations have been extensively discussed, had led to formulate the concept of biomechatronic systems and inspired the work on a *plantoid* robot (Mazzolai et al., 2010, 2014). It was proposed that robots with bioinspired structures or functionalities could be used for testing the biological hypotheses in a more reliable way than using the *in silico* experiments (Mazzolai et al., 2010; Schmickl, 2011; Zahadat et al., 2015). Bioinspired robots with different root-like functionalities have been under development, but until now, a few versions have been presented (Sadeghi et al., 2014, 2016). The non-random root behavior has led to the development of preliminary plant computing system that are based on root logical gates, which are a new development in natural computing research area (Adamatzky et al., 2016). Swarming was proposed as an experiment-based model for coordinated growth of roots (Ciszak et al., 2012; Kawano et al., 2012), and similar models could have an influence on the functioning of future biohybrids controllers. In this article, we focus on possibilities of communication between robots and plants, which emerge from biological processes existing *in planta*. We indicate processes on the level of interorganismal communication (between plants and other plants, animals, or microorganisms) that could be used for the robot–plant biohybrids development. We discuss also different models representing plant development, responses to environment, and structures, which could become useful for the development of controllers for biohybrids. Moreover, we present some of the already available devices, mostly agricultural sensors, which will be useful for robot–plant biohybrids development.

## Tropisms

Tropisms are plant responses to external stimuli, which depend on stimuli’s direction. The tropisms improve plant adaptability by fitting plant growth to environmental constraints and localized resources. Tropisms could become beneficial also for robot–plant biohybrids. One of the proposed biohybrids tasks would be to create growing architectural artifacts, where biological symbionts, as well as robotic partners, collaborate in the process of building a structure (Hamann et al., 2015; Wahby et al., 2015). For this reason, biological processes that enable automated shaping of plants could become crucial for biohybrids functioning.

### Gravitropism

Redirection of growth according to a gravity field is called gravitropism. Usually, shoot grows in the opposite direction to the gravitational force (negative gravitropism) and, on the other side, root shows positive gravitropism. Roots and shoots contain cells specialized in gravisensing, called statocysts. Amyloplasts-statoliths, endomembrane system, and cytoskeleton are involved in gravity perception in statocysts (Mancuso et al., 2014). Further, the formation of auxin gradient determines a plant’s response (Morita and Tasaka, 2004). The polarization of auxin export protein, PIN2, toward root tip was shown to be indispensable for gravitropism (Rahman et al., 2010). The gravitropism is essential for establishing plant’s orientation, but due to difficulties in the manipulation of the gravity field, it probably would not be very useful in shaping especially bigger plants, like trees, within biohybrids.

### Thigmotropism

The thigmotropism is the directional growth in response to touch or contact with an object. Touch can stimulate the fastest known plants’ motions, which are reported for carnivorous plants and *M. pudica* (Braam, 2004). The positive thigmotropism is present in climbers, mostly in specialized structures called tendrils. Tendrils activity result in bending and coiling of the plant around the support, in plants such as vines, ivy, lianas. Auxin is the main phytohormone responsible for tendrils development and movement, but more complex phytohormones interactions were shown so far in *P. sativum* morphogenesis (Braam, 2004; DeMason and Chetty, 2011). Touch is important in the regulation of plant growth and development, this phenomenon is called thigmomorphogenesis, but generally plants rarely show pronounced thigmotropisms in shoots. However, touch and mechanical stimulation lead to the reconstruction of tissue, what changes biomechanical properties, and also leads to activation of defense mechanisms or touch avoidance response (Braam, 2004; Chehab et al., 2012; Badel et al., 2015). In robot–plant biohybrids, touch has to be considered as an important factor that can influence plant reaction to robotic components. Such interaction might result in growth suppression or reorientation, and also in the change of biomechanical properties of tissues. This could become an important process, especially considering living buildings and plants as structural elements. Trees adapt their internal structure of trunk and branches to stress they experience; reaction/compression wood could be formed with different biomechanical and hydraulic characteristic (Badel et al., 2015). Tree structure shows property of embodied memory, what can be exploited with architectural biohybrids.

### Phototropism

Plants depend on sunlight to carry out the various biological processes and in order to adjust growth according to light. The diverse classes of photoreceptors are responsible for sensing the light of different wavelengths. Plant’s photoreceptors are cryptochromes, phototropins, phytochromes, and UVR8, all of them are proteins responsible for many key developmental processes and for adaptation to light conditions (Fankhauser and Christie, 2015; Galvão and Fankhauser, 2015). Cryptochromes are blue light receptors involved in photomorphogenesis: seed germination, seedling development, and transition from vegetative to flowering stage. The another group of photoreceptors are phytochromes, which absorb blue, red, far red light (600–750 nm). In seeds, phytochromes are required regulation of germination, which depend also on red—far-red light balance. The phytochromes help also to adjust plant’s growth according to the changing seasons. UV light in range 280–315 nm is detected by a UVR8 receptor, which is also known as a UV-B resistance receptor. The plants are continuously exposed to the sunlight and UV-B during the day, this radiation could cause photodamage to macromolecules and disrupt photochemical processes. The UVR8 receptor protects plants from UV-B attack by activating genes responsible for the synthesis of protective pigments.

Phototropins, blue light receptors, are the most prominent photoreceptors involved in phototropism. The detection of light by phototropins occurs at the plasma membrane and chloroplasts outer membrane, then the signal is transmitted to other subcellular compartments (Aggarwal et al., 2013, 2014; Kong et al., 2013). There are two genes encoding phototropins in higher plants: phototropin 1 (phot1) and phototropin 2 (phot2). The phot1 is a primary receptor, which controls phototropism under the low intensity of blue light. Both, the phot1 and phot2, act under moderate to high intensity blue light. The phototropins, except phototropism, impact also photosynthetic capacity, photosynthetic gas exchange, and stomatal opening, thus phototropins also adjust metabolism for changing light conditions. The phot2 activation under highintensity light is indispensable for chloroplast dislocation from irradiated side of cell and avoidance of photodamage (Kong et al., 2013). Both the blue light and the red light are important regulators of phototropism; probably, all mentioned photoreceptors can modulate phototropism. Generally, blue light activates phototropic curvature, red light plays a rather additional role in the modulation of phototropic sensitivity. It was concluded that regulated auxin transport is a likely mechanism controlling phototropism (Briggs, 2014; Liscum et al., 2014; Fankhauser and Christie, 2015). Moreover, it was shown that PIN auxin exporters, particularly PIN3 protein, act downstream of light perception to contribute asymmetric auxin distribution to phototropic hypocotyl bending (Ding et al., 2011; Rakusov et al., 2015).

Phototropism is the most promising type of plant tropisms that could be used for manipulation of plant shape in a non-invasive way. Recent knowledge suggests that using different light wavelengths could lead to different results, influencing growth differently. The complexity of plant light-related processes should be taken into consideration and efficiently utilized, to account for light influence on growth direction and on metabolism. Affecting direction of plant growth with light source’s position would have an impact on development and photosynthesis, similarly to pleiotropic effects of sunlight. It also could be predicted that changing light source’s position outdoor would not be fully effective, due to the probable dominant effect of sunlight on plants. For localized induction of phototropism, the most prominent results could be obtained in darkness/dim light, where light that has to stimulate phototropism would be the strongest light reaching a plant. This effect could be achieved by constant illumination of chosen targeted organs, but it could have an additional negative side effect (Velez-Ramirez et al., 2011). The greenhouse experiments might become useful in choosing an appropriate light that plants will favor (Lin et al., 2013; O’Carrigan et al., 2014). Phototropism regulation might be species-specific, the requirement of efficient light stimulation has to be tested according to species and environmental conditions.

## Plant Communication

For many years, the idea of “talking trees” was in contradiction with human common sense. Plants were described as simple living automats, which in the best case could only gather and use information from the environment but could not communicate with other organisms. However, plants are capable of both sensing their environment and actively changing it (Kegge and Pierik, 2010). The first study on plant communication was reported in early 1980 (Baldwin and Schultz, 1983; Rhoades, 1983). A higher level of herbivore resistance was described in conspecific plants growing in close range to herbivore-attacked ones than in plants growing further away. Since then, the concept of plants interacting with each other, as well as with microorganisms and animals, have become well established (Kesselmeier and Staudt, 1999; Dicke and Baldwin, 2010; Holopainen and Blande, 2012; Karban et al., 2014; Mescher and Pearse, 2016; Schöner et al., 2016).

We suggest innate plant communication mechanisms with other organisms and environment as the most convenient approach to establishing an artificial connection between plants and robots. Human ability to transform plant signaling pathways is far more limited than the modification of robots. Hence, adjusting robots for perceiving plants derived signals and transmitting to plants nature-like compounds should become a priority in establishing plant–robot communication.

### Plant Volatiles

The best-described channel of plant communication are air-borne signals conducted by small (<300 kDa) organic compounds with high vapor pressure called biogenic volatile organic compound (BVOC). More than 1,700 such compounds are known, which can be divided into four classes: terpenoids, phenylpropanoids/benzenoids, fatty acid derivatives, and amino acids derivatives (Dudareva et al., 2006; Blande and Glinwood, 2016). Plants emit BVOCs in response to a variety of stimuli: herbivore attack (Kost and Heil, 2006), mechanical damage (Duran-Flores and Heil, 2016), pathogen infection (Yi et al., 2009), and abiotic stresses (Loreto and Schnitzler, 2010). BVOCs are perceived by plants, microorganisms, and animals (Blande and Glinwood, 2016). Volatile compounds can be synthetized *de novo*, as a part of systemic defense response or can be stored in vacuoles or in laticifers and be released while mechanical plant damage and disruption of cellular compartments occur (Holopainen and Blande, 2012). Compounds release and perception depend on the chemical characteristic, and can occur through leaf stomata, the membrane of epidermal tissues or other structures, such as osmophores (Baldwin, 2010).

### Plant–Plant Volatiles Communication

Plants utilize BVOCs to communicate intraspecific—between plants of the same species (Bruin et al., 1992; Engelberth et al., 2004; Karban et al., 2006; Kost and Heil, 2006), interspecific—between plants of different species (Farmer and Clarence, 1990; Karban et al., 2000) and within-plant—between organs of the same plant (Karban et al., 2006; Frost et al., 2007; Rodriguez-Saona et al., 2009) information.

Mechanism of perception on the cellular and molecular levels is still to be unraveled; no specific receptor responsible for the detection of BVOCs have been discovered. However, it has been shown that membrane depolarization and the influx of calcium ions into cytoplasm are important stages of induction defense mechanism in presence of BVOCs (Zebelo et al., 2012). It is proposed that metabolism of received BVOCs in plant tissue is required for efficient perception (Matsui, 2016). A common response of plants to BVOCs is an acquired resistance, which results in reduced damage done by herbivores or pathogens (Niederbacher et al., 2015). Various mechanisms are involved in resistance’s acquisition: production of phenolics or proteinase inhibitors (Farmer and Clarence, 1990; Tscharntke et al., 2001), increased level of phenolicspyrochatechol, chloragenic acid, gallic acid and p-hydroxyl benzoic acid (Hu et al., 2009), activation of genes involved in defense response (Kikuta et al., 2011). Recent studies showed that interplant communication by BVOCs is rather defense priming, it makes plants respond faster and strongly once attacked (Karban, 2015). Plants usually release a specific mix of different VOCs, and their reception by a plant is based on the concentration of various volatiles, and change in this proportion, what could result in a reduction of defense answer (Kikuta et al., 2011). The plant can respond to the received BVOCs also by emitting volatiles of similar composition as in received BVOCs mix. It means that the initial message could be spread over a large area (Giovannetti et al., 2004). It was shown that plants emit BVOCs in reaction to abiotic stress, what was considered as a physiological protection from environmental constraints (Loreto and Schnitzler, 2010). Although, there are very few studies that show plant’s interspecific response to the volatiles compound emitted due to abiotic factors (Yao et al., 2011, 2012).

Within-plant communication using BVOCs is faster than vascular chemical signaling and more independent of anatomy (Heil and Karban, 2010). It was demonstrated that BVOCs released from damaged parts of the plant prime resistance in other organs of the same plant (Frost et al., 2007). Interspecific communication strongly related to herbivore attacks is based mostly on volatile compounds, such as methyl salicylate and methyl jasmonate, which are strong inducers of defense answer regardless of plant species (Tamogami et al., 2008). Due to the restricted BVOCs effective range—10–60 cm (Karban et al., 2003, 2006) and lack of direct benefits for emitter plant, it is considered that all plant–plant communication phenomena are derived from within-plant communication, and other interactions between plants are only “eavesdropping” of this signals (Heil and Karban, 2010). However, on the other side, some reports showed BVOCs effect on neighboring plants up to 10 m, what suggest the inclination to reach neighboring organisms (Tscharntke et al., 2001).

### Plant–Animal Volatiles

Plants do not interact only with themselves; BVOCs are perceived by animals also. The nature of this communication is very complex and reaches beyond third trophic level (Stam et al., 2014). Herbivore-induced plant volatiles (HIPVs) can act as a direct defense mechanism, decrease the fitness of herbivore, or deter herbivore oviposition (De Moraes et al., 2001; Kessler and Baldwin, 2001). Indirect HIPVs-based defense depends on alluring of insectivorous, herbivore parasitoids, hyperparasitoids, and even vertebrate predators (De Moraes et al., 1998; Mäntylä et al., 2004; Rasmann et al., 2005).

### Root-Emitted Compounds

Plants communicate not only through air-borne signals, they emit and perceive signals also underground, because roots can release infochemicals just like the aboveground plant organs do (Peñuelas et al., 2014; Belhassen et al., 2015). It was shown that plants respond to the arrangement of the neighboring plants’ roots (de Kroon, 2007; Ciszak et al., 2012). Depending on the level of relatedness surrounding, plants can avoid neighbor roots, change allocation, modify profiles and levels of secreted proteins (Mahall and Callaway, 1991; Callaway, 2002; Dudley and File, 2007; Badri et al., 2012; Depuydt, 2014). Above- and below-ground herbivores can induce emission of root signals that can be perceived by other plants. As a result, stimulated systemic defense response can be induced, following increased attraction of the predatory to herbivores arthropods or parasitoids (Dicke and Dijkman, 2001; Guerrieri et al., 2002; Cheol Song et al., 2016). Biotic stresses are not sole inducers of interplant communication, in a similar manner, it can be induced by abiotic stresses. Studies show that plants exposed to osmotic or drought stress close stomata and send signals to unstressed neighboring plants, what causes the same behavior, although, for a shorter period of time (Falik et al., 2012). Other organisms could also perceive root-derived compounds such as sesquiterpene (E)-β-caryophyllene, emitted by maize roots in case of larvae-induced damage (Rasmann et al., 2005; Turlings et al., 2012). Also, pregeijerene, secreted by citrus roots upon larval *Daiprepes abbreviates* feeding, strongly attracts an entomopathogenic nematode (Ali et al., 2012). Though the range of this communication is limited to about 10 cm (Turlings et al., 2012).

### Plant Communication through Common Mycorrhizal Networks (CMNs)

Functional communication related to diffusion of signaling substances in the ground is not effective, due to biotic and abiotic degradation, sorption to organic matter, the formation of complexes with metals, and density of the medium (Kaur et al., 2009). This limit is overcome by signaling through underground CMNs. CMNs are formed by arbuscular or ectomycorrhizal fungi and the interconnected networks of fungal hyphae (Selosse et al., 2006). Such network could connect many different taxonomic diverse plants and fungi, over a large area (Giovannetti et al., 2004). Crucial nutrients for plants are transported *via* CMNs, such as phosphorus, nitrogen, and carbon (Selosse and Roy, 2009; Smith and Read, 2010), as well as lipids, phosphate transporter, and amino acids (Bago et al., 2002; Jin et al., 2005). Although, it was shown that CMNs can act as a channel for plant communication too. Infested plants could send signals through CMNs to other plants connected with the same network and induce resistance—the release of BVOCs in a receiving plant (Babikova et al., 2013). Five potential mechanisms of this communication have been proposed: (1) the signal could be conducted *via* cytoplasm or, (2) apoplastic transfer within hyphae (Barto et al., 2012), (3) mycorrhizal hyphae can twine together creating cords with existing plant tissue, forming channels at the interior of the cord filled with water or air (Friese and Allen, 1991; Barto et al., 2012), (4) the message could be conducted by electrical signals as a result of membrane depolarization (Johnson and Gilbert, 2014), (5) infochemicals could be transported in hyphae exterior layer of water *via* capillary force or in microorganisms-dependent way, though close interaction of hyphae with soil makes this route unlikely to function over a large distance (Johnson and Gilbert, 2014).

## Application of Plant–Plant Communication

It has been discussed for years, how to utilize natural plant communication in agriculture, forestry, and architecture. One of the obstacles is a complexity of signal signatures and diversity of possible responses. Since the first studies of the role of volatiles in survival and adaptation, BVOCs has been successfully used in pest insects control (Sobhy et al., 2014) and in establishing crop resistance to abiotic stresses. Possible application of natural plant signals as pest control mechanism have become an interesting solution to the problems related with growth of human population, increasing food demand and well-known toxicity of the synthetic pesticides. At this moment, there is a growing number of “push and pull” approach initial tests (Du et al., 2016; Zhou et al., 2016); however, only one successful implementation was reported at large scale (Cook et al., 2007). A successful method based on the integration of stimuli that act (e.g., BVOCs) to make protected crop unattractive to the pest, while luring pests toward a different target that was chosen by the farmer (Cook et al., 2007). While most of “push and pull” tests utilize natural plant abilities, a first field test using genetically modified plants emitting insect pheromones was also performed (Bruce et al., 2015). Although, approaches to using GMOs in pest control could be more efficient, such as luring insectivorous or parasitoids by plants with an enhanced level of BVOCs emission (Kappers et al., 2005; Kos et al., 2013). Artificial emission of specific volatiles has been also investigated, what suggests possible future utilization of BVOCs in biohybrids (Kelly et al., 2014). It was shown that robotic mechanical wounding could stimulate plant defense mechanisms, like BVOCs emission, although, in a different way than an insect attack. Two applied types of mechanical damages resulted in activation of different types of signaling events (Bricchi et al., 2010). It proves the complex mechanism of regulation of BVOCs emission and suggests that use of artificial tools to create a certain plant response has to be more thoroughly investigated.

Nevertheless, in the perspective of biohybrid-plant communication, these are preliminary, but promising results and actions, which focus on BVOCs in plant communication happening aboveground. Detection of all compounds from BVOCs mix emitted by a single plant is still a very sophisticated process (Materić et al., 2015). Gas chromatography (GC)-mass spectroscopy (GC-MS) is a gold standard in chemical analysis of BVOCs, even very similar compounds can be separated. Even though downsizing is possible (Tridion-9 GC-MS), measurements are far from real-time detection (Materić et al., 2015; Niederbacher et al., 2015). The technique allowing BVOCs measurement in real-time is ion flow tube-mass spectrometry (SIFT-MS), which is direct and quantitative and does not require calibration against gas standards. On the other hand, isoprene and monoterpenes cannot be measured simultaneously, and different isomers cannot be distinguished (Materić et al., 2015). Another possible technique is proton transfer mass spectrometry (PTR-MS), the most sensitive real-time technique, but identification is limited to the nominal mass (Schaub et al., 2010). Although, some modifications of this method or coupling with GC can discriminate single compounds (Misztal et al., 2012). The even more challenging task is the measurement and detection of plant signals underground since the soil is far much more dense and heterogeneous than air, and humidity changes can affect the diffusion and distribution of infochemicals. Recent approach to cope with these impediments is inserting sampling devices in the soil, next to the root, but most of them cannot provide real-time measurement and cannot detect signals transmitted through CMNs (Blande and Glinwood, 2016). Another example of detection technique is bi-enzyme biosensor using carbon nanotubes (CNTs), which is able to detect in nanomolar range methyl salicylate (MeSA), one of the plants common BVOC. This bi-enzyme biosensor consists of electrochemical biosensing platform with immobilized enzymes (salicylate hydroxylase and tyrosinase), as recognition elements. Enzymes are immobilized onto CNTs matrix on dedicated carbon electrode surface. This biosensor selectively detects MeSA, which is known as a potent VOC released by plants mainly under biotic stress. This technique could be applied as a swift method for detection of pathogen infections before harsh phenotypic changes had occurred (Fang et al., 2016a,b). E-noses were also shown to detect and discriminate BVOCs, so multiple technologies could be investigated for being applied as a tool for detection of BVOCs derived from plants in biohybrids (Ghaffari et al., 2012). Such capability of robot–plant biohybrid to detect early infections will help in keeping plant fitness on both, organismal and ecosystem levels.

Although it could seem discouraging in creating plant–robot communication, we do not have to measure and differentiate all BVOCs and other infochemicals simultaneously. We should focus on examined, most important and influential compounds, creating suitable, miniaturized, and real-time sensors. This approach, as well as crucial volatile compounds emission devices could allow to establish a two-way communication in plant–robot biohybrids. The goal is to use robots that could not only detect changes in plants but ones that could also modify plant metabolism, development, and defense response through mimicking and amplifying natural plant signals. Bearing in mind that robots could communicate and coordinate actions on a relatively large area, it could provide a great tool to control not only single plant, but to govern a whole field or ecosystem at the same time.

## Long-Distance Signaling

Plants, although immobile and not moving rapidly, can react quickly to changing environmental conditions. Information about some of the environmental changes that are perceived locally has to be transmitted to other distal organs to allow for an adaptation. Attack of a herbivore or a mechanical damage caused by the wind are examples of events that require a fast reaction on the local and whole-organism levels. Different types of rapid long-distance signaling pathways ensure an efficient communication between distal organs and make it possible to develop a complex system of “signatures,” which can lead to a proper response to a stress factor. Deciphering plants’ reactions that emerge rapidly after stimuli would offer a chance for quick adaptation of robotic elements of biohybrids to changes in the environment and to plant-counterpart’s physiological conditions.

### Hydraulic Signals

Hydraulic signals, relatively simple by nature, but still not extensively explained, can be transferred theoretically even at a sound’s speed in plant’s xylem vessels. Till now, speed ranges of 0.03–20 cm*s^−1^ were reported in experimental works (Huber and Bauerle, 2016). Hydraulic signals spread information about changes in water balance and vessels status, what induces a positive or negative alteration in xylem tension. Positive tension alteration can be caused by heat, fire, mechanical bending, however, not all causes are thoroughly explained. Especially, the correlation between cavitation, drought, and corresponding hydraulic signals, is still to be investigated. Negative alteration in the xylem tension occurs due to drought and salt stress. Hydraulic signals are perceived by parenchymal cells, but no further signaling events are known, although mechanosensors have to be involved (Christmann et al., 2013). The cross-talk of hydraulic signals with a different type of signaling is elusive, except the known strong connections with abscisic acid (ABA) signaling (Christmann et al., 2013). Even though sap flow or turgor sensors could be used in potential biohybrid as sensors of hydraulic signals, the current status of biological knowledge does not allow for reliable interpretation of detected signals.

### Chemical Messengers

Chemical messengers, like phytohormones, proteins, peptides, RNAs, could be transported for long distance in phloem or could act as a global gradual metabolic response (Xia et al., 2015). Especially, plant phytohormones, like auxins, cytokinins, gibberellins, ABA, ethylene, jasmonic acids, stringolactone, brassinosteroide are known to be crucial for controlling plant’s physiology. Detection of phytohormones level would be beneficial to estimate plant’s condition in biohybrids and to predict future growth and development. On the other hand, application of phytohormones within biohybrid could help to control plant’s physiology, obtain “programmed” shape/architecture, increase plant fitness. Phytohormones are intensively used in agriculture and potentially could become one of the most important substances by which biohybrid robots would monitor plants and which robots would use to affect plants. It has to be mentioned that phytohormones induce complex signaling pathways and the diverse effects on growth and development, hence, interpretation of detected phytohormones level would be non-trivial. Because of this, an efficient plant’s model including the dynamic interplay of different phytohormones (Xia et al., 2015) is required. Moreover, considering methodology, non-invasive analysis of phytohormones levels in a plant tissue could not easily be performed with available technologies. Chromatographic methods are standard, but they are challenging even in lab conditions (Almeida Trapp et al., 2014). Also, application of phytohormones would depend upon the dosage system, what could become an obstacle in a biohybrid’s functioning in long timescale in a field or urban space.

Proteins and RNAs, that also determine plant growth and development, are transported from an organ to organ by plasmodesmata or by vascular tissues. The fate of a cell is determined mostly by transcriptional reprogramming, so detection of mobile transcription factors transport would be very interesting. For example, it was shown that HY5 transport from shoot to root is required for optimization of carbon and nitrogen uptake (Chen et al., 2016). Detection methods for macromolecules, ELISA for proteins, PCR for nucleic acids, are now well established in laboratory conditions, but further miniaturization and automatization would be required for implementation of them within biohybrids. Moreover, due to the need of reagents exchange in every reaction, it would be difficult to apply these methods within a long-time running robotic device.

### Reactive Oxygen Species (ROS)

Reactive oxygen species were discovered as destructive molecules, but nowadays, it is established that they are critically important for both intra- and intercellular signaling and are broadly involved in stress response and plant development. In roots, ROS were shown to be involved in regulation of a cell cycle, cell differentiation, root hairs, and lateral roots development—the importance of ROS regulatory role is comparable with phytohormones (Tsukagoshi, 2016). ROS are side products of oxidative processes in chloroplasts, peroxisomes, and mitochondria, but ROS are also generated by specialized enzymes, like RBOHD. Respiratory oxidase homolog D (RBOHD) is a protein hub for calcium, electric, NO, MAPK, and ROS signaling pathways (Batistič and Kudla, 2012; Xia et al., 2015; Gilroy et al., 2016). Considerable progress in understanding of ROS role has been recently obtained and the image of complex signaling network has emerged (Gilroy et al., 2014, 2016; Xia et al., 2015; Noctor and Foyer, 2016). For biohybrids, measurement of ROS or detection of ROS wave, i.e., ROS rapid long-distance signaling, could result in obtaining valuable biological information in relation to spatiotemporal data. From a methodological point of view, the detection of ROS depends mainly on staining and imaging, which would not be easily applied in biohybrids (Steffens et al., 2013). Interestingly, constant development of electrochemical methods of H_2_O_2_ detection in plant cells should be mentioned (Olvera-González et al., 2013; Prasad et al., 2015). Probably, they are more applicable in biohybrids, for they omit microscopic image acquisition and processing. However, the calibration and decoding of correlation between spatiotemporal data, biological processes, ROS levels, and changes should be performed at first.

### Calcium Waves

Changes in calcium concentrations in different subcellular compartments and propagation of calcium waves along cells’ plasma membranes are key events that determine an answer to changing conditions and influence the cell’s further fate (Batistič and Kudla, 2012). Calcium wave is triggered rapidly after a stimuli’s perception and leads both to genetic reprogramming and to a signal propagation. Subcellular calcium concentrations and oscillations lead to different calcium signatures that are recognized by specialized protein, like CaMs or CDPKs, which trigger downstream events (Steinhorst and Kudla, 2014). For example, calcium oscillations and following cellular signatures had been described with great details for initial phases of a plant-fungus symbiosis (Chen et al., 2015; Liu et al., 2015). Calcium intercellular waves are strongly integrated within the rest of known long-distance signaling mechanisms and are crucial for cross-talks between different signals (Gilroy et al., 2016), hence calcium is an attractive target for robotic sensors. Imaging methods for calcium detection are hardly applicable in biohybrids and also electrode measurement of calcium cell concentrations is challenging; however, Ca^2+^-specific electrodes exist and could be investigated more with regards to this purpose (Brownlee, 1992; Batistič and Kudla, 2012).

### Electric Signaling

Electric signaling in plants is a quite intriguing physiological process, which provokes scientists to talk even about plant neurobiology (Brenner et al., 2006). For robot–plant biohybrids, electric signaling is a promising way for plant–robot communication, due to their analogy to successful technologies applied in the robotic prosthesis. The most prominent example of plants electric signaling is actions potentials (APs), which trigger closing of *D. muscipula’s* trap (Volkov et al., 2013; Böhm et al., 2016). Three or even four types of electric signals were discovered in plants: APs, variation potentials (VPs), system potentials (SPs), and possibly wound potentials (WPs) (Gilroy et al., 2016; Huber and Bauerle, 2016; Zimmermann et al., 2016). Because of lack of cell–cell electrophysiological connectivity, like “neuron-highways,” APs could hardly be propagated for long-distance, except in a few plants, like *Dionaea*. VPs also seem to be unable to self-propagate for long-distances. Only SPs, which depend on H+-ATPase activity are self-propagated for long-distances and lead to plasma membrane hyperpolarization (Zimmermann et al., 2016). Electric signals speed range from 0.08 cm*s^−1^ for SPs to 20 cm*s^−1^ (or even 4,000 cm*s^−1^ in shown in Glycine max) for APs. APs were induced with cold/ice stimulation, VPs, or SPs rise after wounding (Fromm et al., 2013). Involvement of electrical signals in different types of biotic and abiotic stresses was shown in multiple research (Fromm et al., 2013; Mousavi et al., 2013; Ríos-Rojas et al., 2014; Zimmermann et al., 2016). In an interesting study, the specific occurrence of VPs after chemical herbivore-derived treatment, differently than in the case of robotic arm-derived mechanical damage (single or repetitive herbivore-like mechanical damage) was shown (Bricchi et al., 2010). Moreover, the nature of biotic stress influences the time of plasma membrane depolarization and subsequent change in genes expression, but not the depolarization characteristic (Bricchi et al., 2012).

It has to be highlighted that detection of mentioned signaling and molecules is a very challenging task, but it would ensure tight connections with plants biological processes and pathways that further determine the developmental decision. Preliminary studies on plants electrostimulation were conducted and interesting characteristic of plants electric properties have been revealed (Volkov et al., 2013, 2017; Volkov and Shtessel, 2016). Close links between membrane potential changes, calcium waves, ROS signaling, suggest that monitoring of even three types of signaling pathways would be the most reliable in deciphering the biological meaning of long-distance signals. However, a long-term measurement of signals of one type, but in different organs, would be also very helpful for a plant–robot communication. Understanding of plant electric signaling enables not only investigating a plant’s condition but also could allow for efficient influencing of the plant’s growth, development, and adaptation by application of voltage targeted into tissues. Moreover, data obtained during biohybrid work could be beneficial for plant science and our understanding of plants.

## Sensing Devices—On the Way to Biohybrid

Information flow from plants to robots could depend on diverse plant processes, some of which could be monitored by wet-lab analysis or use of electronic sensors. Laboratory-like actions are not easily applicable in biohybrid conditions. Sensors that would be able to measure plant’s relevant physiological variables may be used for collecting data from plants in biohybrids. This becomes very useful for establishing a communication channel between artificial and natural elements. It is important that only non-destructive methods of measurement of physiological processes should be considered; invasive, destructive methods could influence plants in many undesirable ways (Busemeyer et al., 2013; Tattaris et al., 2016). There are many sensors available on market, which enable to measure different agriculturally relevant plant features. Decagon Devices (now Metergroup) company is specialized in sensors, which are designed to collect data from the forest canopy. Ready sensors allow, for example, to measure leaf area index (LAI) (Sone et al., 2009), stomatal conductance measurements, electromagnetic radiation reflected from canopy surfaces, leaf wetness, tree or branch circumference, surface temperature, and more. There exist also a ready microclimate monitoring station and a set of sensors, which deliver information about the soil, and irrigation. Yara Water-Sensor monitor water status of the crop by measuring the turgor pressure in leaves, hence it helps in irrigation’s automatization and could be used even to detect hydraulic signals (Yara, 2017). Data collected from these sensors, together with climate and soil data may strongly facilitate designing, development, and implementation of electronic elements in robot–plant biohybrids. Such sensor system would enable the reliable observation of robot’s impact on plants, one of the key feature for a working biohybrid. Development of biosensor plants in order to show changes in physiological status of plants in a way that would allow for easier data collection, for example, luminescence or color change, could open new possibilities (Feng et al., 2015; Medford and Prasad, 2016). Adaptation of some already existing laboratory methods to requirements of biohybrids could also allow for progress in the future.

One of the tools that could be used for organ specific delivery of effective compounds to plants are organic electronic ion pumps (OEIPs), a polymer-based delivery system and organic electrochemical transistor (OECT) (Simon et al., 2009a; Nielsen et al., 2016). They can deliver with high precision and efficiency a range of biologically relevant substances. Ion transport was shown to be able to mimic, and to precisely affect Ca^2+^ signaling in a programmed way (Simon et al., 2009a,b). It was demonstrated that OEIPs can be used as programmable “machine-to-brain” (or “machine-to-cell”) communication platform. Recent studies have shown that it is possible to couple OEIP with stimulation unit—a sensing unit, which may work in parallel (Jonsson et al., 2016). There are many more variants and different approaches to signal transfer—organic thin film transistors (OTFTs), polymer electrodes, smart textiles, ion bipolar junction transistors, chemiresistors—which could be applied to develop biohybrids. For example, OTFTs may be used as sensors for: pH, K^+^ ions, cysteine, glucose, lactate, DNA, humidity, and saline (Liao et al., 2015). It was also shown that in fixed plants xylem, a transport tissue in plants may be used as a wire to conduct electronic signals delivered by OECT (Stavrinidou et al., 2015). Nowadays, one of prominent research trend is to fit these devices for applications in neuroscience (BiOprobe Project, FP7-PEOPLE, Project ID: 300106).

Programmable ligand detection system in the plant, based on periplasmic-binding proteins and ligand receptors, was used to develop highly sensitive and specific sensor system (Antunes et al., 2011). These precise receptors, linked with plant visual response system (e.g., bleaching of the plant in the case of detection of ligand or luminescence), could be used for simple and safe detection of many receptor ligands—chemical agents, volatile compounds, or even explosives (like TNT—2,4,6-trinitrotoluene). It was shown that it is possible to develop transgenic *A. thaliana* and *Nicotiana tabacum* (called “detector plants”), which are capable of detecting TNT by roots and shoots. The response of plants to detection of ligand (de-greening) was visible after 24–48 h (Antunes et al., 2011). Recent studies have shown that wild-type plants can be also considered as pre-biohybrids—by using nanoparticles. Nanobionic-plant system was created; it is based on spinach plant *(Spinacia oleracea*) and nanoparticles—single-walled carbon nanotubes (SWCNTs) (Wong et al., 2017). This system was able to detect nitroaromatics in real-time and subsequently report that *via* attenuation of near-infrared (nIR) fluorescence in leaves. Nitroaromatics are transported from roots to leaves and then are aggregated in leaf tissue. That process results in relative changes in nIR emission intensity—which could be observed in real-time by nIR fluorescent nanosensors—SWCNTs (Wong et al., 2017). Using SWCNTs opens a lot of possible ways in the design and implementation in robot–plant biohybrids. New functionalities could be added, which will help in obtaining plant’s physiological status or environmental traces, like the presence of TNT.

Design and development of devices called electronic nose (e-nose) could also be useful in creating of biohybrid’s devices. There are a few examples of ready e-nose devices on the market. One of them is zNose^®^, which could analyze and identify traces of organic compounds and can be followed by GC to improve the accuracy of the analysis (Electronic Sensor technology Inc., 2017). E-nose developed by NASA (called JPL Electronic Nose—ENose, third generation) is used currently on the International Space Station to monitor the environment in laboratories and detect air contamination from spills and leaks. The e-nose device based on multichannel quartz crystal microbalances (QCM) was also demonstrated; it contains polystyrene molecularly imprinted polymers layers as a recognition material. This device precisely detects terpenes emitted from fresh and dried plants from *Lamiaceae* family, what was demonstrated for monitoring of herbs freshness (Iqbal et al., 2010). E-nose device might be upgraded with various modules, for example, vulnerable thermometer, which eliminates temperature impact on measurements (Lin et al., 2016).

The diversity of known technologies to measure various plants traits looks promising. Biohybrids composed of plants and robots will be equipped with reliable sensors, often previously tested in agriculture. Further development of technologies will be greatly appreciated: new sensors, alike synchronization of known sensors into one sensing system, will make it easier to formulate conclusions about plant’s physiological status. Different methods, for example, machine learning or regression analysis, could be then used to extract information about vegetative status (Doktor et al., 2014).

## Mathematical Models of Plants in the Context of Biohybrids

Mathematical modeling fits into robot–plant biohybrid subject as an excellent tool for relatively fast prediction and validation of stimulants that would allow plants to grow in the desired manner. Robots need controllers, which could partially depend on plant’s model to achieve plant related targets, like shape, adaptation, size. Plants display numerous features that can be mathematically characterized, such as a number of leaves, petals, branches, etc. (Battjes et al., 2016). It seems that significant mathematical correlations can be found by the study of development and plant response to environmental cues at both structural and molecular level (Coen et al., 2004; Chew et al., 2014). At the heart of every computational model is an algorithm—a formula that describes step by step operations that have to be performed in order to depict growth patterns or simulate plant response to different stimuli (Françon, 1997). The main challenge in creating such mathematical model of a plant is to find an appropriate description of the local states that are changing in time (due to, e.g., cell division) and global alterations in the structure of the plant (Prusinkiewicz and Runions, 2012). From a technical point of view, one of the most important breakthroughs in the field was the introduction of so-called L-systems, which is widely used method describing linear and branching structures, where each element is coded as a symbol and all branches are marked in the form of brackets (Janssen and Lindenmayer, 1987). To better describe the system, each element might be bound to other features like length, diameter, or concentration of hormone.

Description of growth and shape itself requires quantitative description expressed in numbers denoting distances, angles, areas, and volumes, while, for example, the arrangements of the elements on the axis can be described without specific measurements (Pabst and Gregorova, 2007). Therefore, one of the most important features that needs to be taken into consideration, when constructing a model, is topology and geometry.

Topology is the arrangement of the various elements in a given system that can be mathematically shown in relations between algorithms describing each element. Although this feature remains constant under different stimuli, while geometric parameters are changing, it is very important to use appropriate topology in the model because the placement of elements later determines exact direction of information flow (Kholodenko et al., 2012).

Model of the overall growth of the plant can be created by integrating all parameters describing local growth (for example, branches, leafs, roots) with a use of tensors expressing length, area, and volume (Jensen and Fozard, 2015). While modeling growth, it is important to consider two viewpoints: the Eulerian and Lagrangian. The first one specifies the region of growth with relation to the external fixed system, while the second one specifies the growth of material element referring to its final version (Prusinkiewicz and Runions, 2012).

Changes in the shape of a certain plant can be caused by the growth of cells, their movement, and death that can lead to the arrest in plant growth (Steeves and Sussex, 1989). Therefore, modeling cell structure and its division is a first step toward elucidating more complex models at the tissue or whole plant level, such as the arrangement of branches, root development, forms of leafs. After integrating all this information, it will be possible to make conclusions about general growth of a given plant in specific conditions.

Plant growth regulation is a multi-level mechanism connecting molecular, cellular, organ, tissue, whole plant, and environmental level processes. Therefore, it is extremely difficult to elucidate exact stimuli that would allow the robot to control plant growth while maintaining symbiosis with it (De Vos et al., 2012). As was mentioned before, the starting point for studying growth in plants are cell pattern, division, and expansion growth models. From that base, every other higher level computational representation could be drawn (Prusinkiewicz and Runions, 2012). This is the reason that reliable model that describe even the growth of a single cell are required.

Plant cells are characterized by the presence of the cell wall. Moreover, they are deprived of cilium or flagellum, hence immobile, so the modeling of expanding growth is connected only to the change in size and shape. In order to model the expansive growth of cells, the equations need to be linked to biophysical processes. One of the most important factor is the turgor of cells, which is connected with water uptake and pressure against plasma membrane. If water uptake is perturbed, a cell’s wall deformation occurs. Hence, cell growth depends a lot on water uptake and transpiration rate on the organismal level (Ortega, 1985, 2010). The latest model explaining expansive growth is LOS model, which is strictly connected with the loss of stability theory. The assumption is that the turgor pressure is decreasing over time to a certain value after the decline of cell wall’s stability (Wei and Lintilhac, 2003). Another recent model incorporates calcium ion movement and chemistry of the cell wall component and suggests that cell wall expansion depends on the rate of the delivery of the substrate for constructing cell wall and pressure level (Proseus and Boyer, 2006).

The process of cell division was discussed as early as in the XIX century. At first, it was suggested that dividing wall was placed at a specific angle along the cell, then it was believed that dividing wall is actually meeting side walls of each cell at right angle, and then it was postulated that the shortest partitioning wall between two cells create two equal cells as a result of division (Sahlin and Jönsson, 2010). Computational models were created based on those assumptions, what showed that all previously suggested rules are needed, but their individual explanatory power is not enough (Sahlin and Jönsson, 2010; Besson and Dumais, 2011).

The right placement and orientation of division walls are the most important for tissue development models. Cells in those models can be represented as a set of points and growth simulated by addition of new points. A division that can occur in that model depends on the size of the structure created after several rounds of new points addition (Korn, 1969). In more recent models, cells in tissues are depicted by series of points with the application of the rule that allows for a random choice of division walls when they have a similar length (Dupuy et al., 2010; Besson and Dumais, 2011). Tissues topology, instead geometry, also can be taken into consideration, due to cells divide with regard to time rather than shape or size, what affect how combinations of L-systems can be implemented (Kennaway et al., 2011; Robinson et al., 2011).

One of the important geometric features of the plant structure is phyllotaxis. The geometric patterns that plants display with their leaves, shoots, or roots are originated from the specific placement of primordia (Braybrook and Kuhlemeier, 2010). What is interesting, angles between following primordia often assume the value of the golden angle (137.5°) or Lucas angle (99.5°) (Prusinkiewicz and Runions, 2012). An important question to be answered is how the positioning of primordia is determined, and then, how the initiation of development of specific tissues occurs. In the first model of this process, it was assumed that already existing primordia may inhibit the development of other initial primordia. Therefore, in the model, authors surrounded each primordium with inhibition zone and new structures were only allowed to develop when there was space for them (Mitchison, 1977). In following models, the distances between primordia and inhibitory effects of other primordia were taken into consideration. New structures were created after those effects weakened with time (Douady and Couder, 1996; Smith et al., 2006). All of these models were effective in recreating of organizations of primordia, while maintaining the golden angle between them.

### Geometric Modeling of the Branching Architecture

In the history of modeling of branching architecture, some of the models take into consideration the morphogenetic role of the environment and others focus only on the internal control of this process. L-systems are used in the models that exclude the influence of environment. Those models allow for the introduction of signals (such as auxin distribution) that can control branching (Janssen and Lindenmayer, 1987; Prusinkiewicz et al., 2009). Others include the more prevalent role of the external control. Those models focus on competition for light and space that occurs between branches. Models of plant development relying only on competition for space show that it is a sufficient factor in creating tree structures (Runions et al., 2007). Although the comprehensive model of branching structure certainly requires incorporation of molecular, as well as, mechanical control of this process (Jirasek et al., 2000; Allen et al., 2005; Cieslak et al., 2011).

### Forms and Development of Leaves

The development of a specific pattern of leaves arrangement is also due to the inhibitory regulation of one primordium over another as a result of auxin distribution. First geometric models of leaves were shown in 1981 and were focused on the propagation of the margin. In the model from 2004, authors introduced specific morphogens that would control the rate and direction of growth. The regulation was expressed in the form of a triangle, whose integration would lead to the leaf surface growth determination (Coen et al., 2004).

### Root Development

The growth of root is connected with a specific concentration of auxins in the apex, as well as with the geometry of the whole root. First models of root development were based on auxin recycling mechanism, where auxins flow to the root apex in the subepidermal layer and away from roots in epidermis from where it leaks again to subepidermal layers, and the cycle is closed (Grieneisen et al., 2007; Stoma et al., 2008). Then, the models of roots development’s initiation were created. These models assumed that development of the root is initiated when auxins concentration reaches a specific limit (Lucas et al., 2008). Moreover, the accumulation of auxins in the initial state provides inhibition of growth of other roots maintaining specific, mathematic pattern. Lateral roots are predominantly created for the upper site of the curved root because those cells accumulate more auxins and the accumulation is mediated by the activity of AUX/LAX proteins (Laskowski et al., 2008).

## Modeling Plant–Environment Interaction

If we consider a plant in its environment as a system, then the phenotype can be described as functional equations multiplied by parameters of the model and external variables in the system (Viaud et al., 2017). Light is one of the most important factors controlling plant’s growth. First simulation models taking into account the environment and light appeared in 1990s (Kanamaru et al., 1992; Měch and Prusinkiewicz, 1996). In those papers, authors proposed the construction of two separate models (plant and environment) and then simulating interactions between them. Communication between plant and environment was set up in the way that the plant sends information about the position of every leaf cluster and its radius and environment reacts to this by incorporating this radius into the light flow. In order to refer to the amount of light in the system, authors calculated the specific quantity of light that reaches plant (Kanamaru et al., 1992). Moreover, the plant model contains a few additional parameters determining the length of branches, the radius of leafs, etc. The simulation starts with a single branch, which supports leaf with an apex. Competition for light is manifested in two ways: either there is reduced branching or arrest in apices development—nevertheless, there is always a reduced amount of branches. In these models, plant responded to the amount and direction of light, which simulate heliotropism, but another stimulus could also be considered (Medina-Ruíz et al., 2011; Viaud et al., 2017). Modeling plant’s response to stimuli is more and more feasible due to the accumulation of plant science’s knowledge, hence, quantitative prediction of when and how a plant responds to different stresses is also becoming more reliable.

Mathematical models allow for prediction of the effect of certain stimuli. Simulation-based models specifically enable researchers to observe the effects on multiple levels of system organization (from molecular to tissue development) in regard to time. After knowing how the cells divide and grow into tissues, it will be possible to model plant organs such as leaves, branches, and roots. This scientific, mathematical approach to plant sciences will make possible to develop biohybrids controllers that could response to plant-derived information in a meaningful and reliable way. On our way to constructing biohybrids, not only engineering should provide more advanced tools but also biology should provide more understanding of plants biology, in a way that could be implemented within applied sciences.

## Recent Developments

Except mentioned earlier attempts and works on subjects associated with plants biohybrids, there are a few EU funded projects that are dedicated for plants bioinspired or plants biohybrids tasks. Two already finished EU funded projects are good example of pioneering and interdisciplinary work on plants and robots. PLEASED project (FP7-ICT, no. 296582) based on idea of using plants as biosensors in non-laboratory conditions, where measurement of changes in plants electric activity were tried to be deciphered and equate to the external events. Estimators for light stimulus on the base placed in plants electrode signals were developed and analyzed (Chatterjee et al., 2014). PLANTOID project (FP7-ICT, no. 293431) was focused on abstracting roots properties that enable effective functioning, and then bioinspired robots were made that could be used for testing biological hypothesis (Mazzolai et al., 2010; Sadeghi et al., 2014, 2016). The PLANTOID’s idea of “plants as robots and robots as plants” is still being intensively investigated and next outcomes and applications are expected. *Flora robotica* (Horizon 2020, no. 640959) is a visionary project aiming to create a highly integrated, symbiotic system of plants and robots in which robots enhance and control the development of plants. The main task is to construct physical structures, with further applications in architecture, resulting from plant growth controlled by robot-delivered stimuli (Hamann et al., 2015). The *flora robotica* is focused on interactions of robots and plants in order to create highly integrated robot–plant symbiotic system. Construction of robot–plant biohybrids, in which robots could guide the direction of plants growth and plants would provide growing structure to robots, is the main of the project’s goals: “the natural plants provide growth structures and sensation capability, robots provide extended sensing and decision-making capabilities” (Hamann et al., 2015). The tropisms were proposed to be used for shaping the plants in the desired way. For example, control by applying LED light over the growing tips of beans was shown, i.e., the directing of plant’s growth to achieve desired spatial targets. The RGB LED light was provided with different settings in order to evolve controllers more efficient in directing bean’s tip into the desired position. These results give background for the further development of biohybrid controllers, which will enable to shape plants (Wahby et al., 2015). Physiological sensors and structural elements that could help in creating robot–plant biohybrids are also under development.

When including also natural computing and part of nanotechnology research, these are signs of growing scientific community that is interested in plants biohybrids related subjects. However, time will tell us which technologies and ideas occur to be the most fruitful and which will be continued—recent state of the art does not allow us to make great prediction.

## Summary

**Figure 1:**
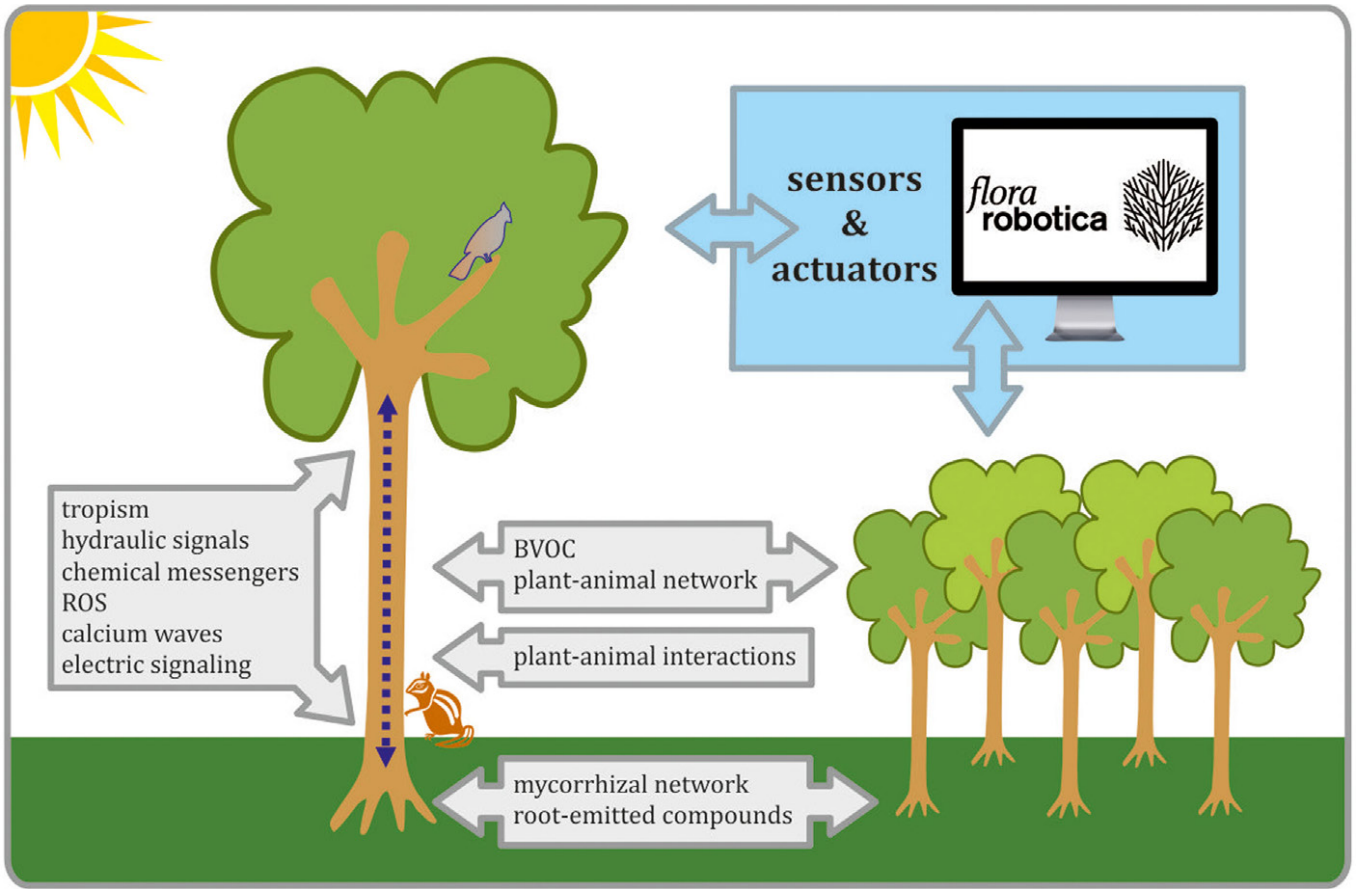
The schematic view on biological process that are important for establishing plants–robots communication in biohybrids. Organismal and interorganismal signaling pathways are considered as crucial in biohybrid development, as well as progress in the field of advanced sensors, actuators, and controllers. All these tasks are undertaken in *flora robotica* project.

Advanced electronics in agriculture, science-based ideas concerning plants’ sensibility, a search of environment-friendly technologies, are all facts supporting the growing interest in robot–plant biohybrids. Living buildings and advanced agriculture are two examples of application of such hybrids that meet global challenges. In robot–plant biohybrids, the artificial part will detect significant changes in both plant physiology and the environment, but it will also purposefully manipulate the plant’s inner processes. We discussed the biological background for establishing plant–robot communication in Figure 1. Conclusions from an analysis of plant biology might direct further efforts concerning biohybrids. Such hybrids would function probably more on the “ecosystem” level and will depend on a massive cooperation between multiple plants/robots. Difficulties arising from decentralized regulation of plant growth and development and the lack of plant nervous system urge us to suggest various possibilities for gathering plants’ data and for influencing physiological processes. However, plants dissimilarity from animals does not change the abstracted life features. Moreover, molecular processes occur very fast and we should try to efficiently interfere with them, if we want purposefully influence decision-making of a living entity (Brunk and Rothlisberger, 2015; Schmickl et al., 2016). Relying on plants macroscopic feedback, which can take days or weeks (like developmental/growth events), could be risky, due to time that enables the appearance of many unpredictable factors that irreversibly influence the organism. Hence, sensing of plants’ biological processes should rely on early and key signaling pathways, which might potentially be used in biohybrids. Similarly, known channels for plant-other organism communication, like volatile compounds, should be exploited. Technological advancements are the keys for the future application of current biological knowledge for establishing plants-robots societies. Sensors will ensure the important link between the world of living organisms and artificial electronic robots. Needs for setting robot’s controllers in biohybrids might enforce implementation of some of the existing plant’s biomechanical or developmental models. Although the creation of robot–plant biohybrids appears very challenging, even now, we could underline some of the plant features that will be crucial for the following steps. As an example, plants ability to communicate with other organisms gives a base for establishing a plant–robot communication channels. Moreover, future developments will not only provide new technologies, but coincident use of biological models, advanced sensors, and methods for manipulating plant’s growth and development might lead to new discoveries concerning plants biology. Despite many challenges to overcome, the robot–plant biohybrids probably are to be soon intensively developed and investigated.

## Author Contributions

TS contributed to writing, correcting, and editing the paper. RK, WK, SW, AS contributed to writing the paper. PW contributed to supervising and correcting.

## Conflict of Interest Statement

The authors declare that the research was conducted in the absence of any commercial or financial relationships that could be construed as a potential conflict of interest. The reviewer LB and handling editor declared their shared affiliation, and the handling editor states that the process nevertheless met the standards of a fair and objective review.

## Footnotes

^1^Heinrich, M. K., Hofstadler, D., Wahby, M., Soorati, M. D., Zahadat, P., Ayers, P., et al. (2017). Survey on constructing living buildings: challenges for a novel application of bio-hybrid robotics. Front. Bioeng. Biotechnol (Unpublished Manuscript).
